# Convention

**Published:** 1889-08

**Authors:** 


CONVENTION for the revision and publication of the Pharmacopœia of the United States of America. We invite attention to the call for a General Convention to assemble in Washington, D. C., at noon of May 7th, 1890, for the purpose of providing for a revision and publication of the Pharmacopœia of the United States of America; and to request that every incorporated medical or pharmical College, Association or Society desiring to be represented in the convention will send its Corporate Title and a list of its officers addressed to the care of Dr. Edwin H. Brigham, Assistant Librarian of the Boston Medical Library, 19 Boylston Place, Boston, Mass.

